# *Staphylococcus epidermidis* ST2 strains associated with bloodstream infections contain a unique mobile genetic element encoding a plasmin inhibitor

**DOI:** 10.1128/mbio.01907-24

**Published:** 2024-11-19

**Authors:** Amy A. Gomez, Clara Kjerfve, Minseo Choi, Wen Liu, Kelly Churion, Sheila Thomas, Holger Rohde, Sam Shelburne, Jon T. Skare, Magnus Hook, Srishtee Arora

**Affiliations:** 1 Center for Infectious and Inflammatory Diseases, Institute of Biosciences and Technology (IBT), Texas A&M Health Science Center, Houston, Texas, USA; 2 Institute for Medical Microbiology, Virology and Hygiene, University Medical Center Hamburg-Eppendorf, Hamburg, Germany; 3 Department of Infectious Diseases, Division of Internal Medicine, Infection Control, and Employee Health, MD Anderson Cancer Center, Houston, Texas, USA; 4 Department of Microbial Pathogenesis and Immunology, School of Medicine, Texas A&M Health Science Center, Bryan/College Station, Texas, USA; Duke University School of Medicine, Durham, North Carolina, USA

**Keywords:** *Staphylococcus epidermidis*, bloodstream infections, mobile genetic element, integrated conjugated element, plasmin, coagulation pathway, molecular pathogenesis

## Abstract

*Staphylococcus epidermidis*, a common commensal bacterium, is a leading cause of nosocomial catheter-associated bloodstream infections. *S. epidermidis* sequence type 2 (ST2) is specifically recognized globally for causing invasive disease. In this study, we identified a novel putative integrated conjugative element, pICE-Sepi-ST2, unique to the genomes of *S. epidermidis* ST2. Our investigation identified pICE-Sepi-ST2 in all ST2 isolates from bloodstream infections. Meanwhile, ST2 isolates from other infection sources, such as catheters, prosthetic joints, and fracture fixations, showed variable pICE-Sepi-ST2 prevalence. pICE-Sepi-ST2 encodes two putative cell wall anchored proteins that we have designated SesX and SesY. Biochemical characterization of SesY revealed that it binds both plasminogen (Plg) and plasmin (Pln) and inhibits Pln’s ability to cleave a chromogenic substrate and degrade fibrin clots. Furthermore, all ST2 isolates containing a pICE-Sepi-ST2 also have a mutated *sdrG* gene. Thus, all ST2 isolates have two genetic modifications that target distinct steps in the hemostatic pathway. SdrG, which inhibits coagulation, is inactivated, and SesY, which inhibits fibrin, is introduced. These findings suggest that the hemostasis pathway is a strategic target for ST2 *S. epidermidis* bloodstream pathogenesis.

**IMPORTANCE:**

This study uncovers a new virulence mechanism in *Staphylococcus epidermidis* ST2 bloodstream isolates. We identify a mobile genetic element (MGE) characteristic of an integrated conjugated element (ICE). pICE-Sepi-ST2 carries the genetic information needed to produce a cell wall-anchored (CWA) protein called SesY. The results indicate that SesY binds to plasminogen (Plg) and plasmin (Pln) and inhibits Pln’s degradation of fibrin clots. Genetic analysis showed that all ST2 bloodstream isolates can express the plasmin inhibitor SesY and carry a mutation in the SdrG gene, resulting in the expression of inactive SdrG. Thus, we describe a molecular pathway targeting the coagulation pathway that may be required for *S. epidermidis* ST2 to cause bloodstream infections.

## INTRODUCTION

*Staphylococcus epidermidis* historically has been considered an apathogenic commensal bacteria colonizing human skin. *S. epidermidis* is now recognized as one of the leading causes of nosocomial bloodstream infections (1, 2). Multiple sequence types (STs) of *S. epidermidis* colonize human skin, but only a small subset of these STs are responsible for invasive infections (3). ST2 is recognized as a global cause of *S. epidermidis* bloodstream infection (1, 2, 4). Numerous studies have attempted to identify features that allow some *S. epidermidis* strains to cause invasive infections. However, no clear answer has yet to be obtained (5–9). Phylogenetic analyses based on core genomes have shown that the *S. epidermidis* isolates from bloodstream infections can be differentiated into three clades but fail to differentiate between invasive and non-invasive isolates (3, 6, 10). Therefore, we hypothesized that the genetic factors that enhance the virulence potential of *S. epidermidis* bloodstream isolates are encoded in mobile genetic elements (MGEs).

MGEs often carry genes for virulence factors that enhance the microbe’s survival in different environments (10). Examples of virulence factors in MGEs include cell wall-anchored (CWA) proteins, antibiotic resistance determinants, heavy metal resistance determinants, and immune evasion factors (10, 11). CWA proteins are often essential virulence factors of Gram-positive pathogens (12–14). MGEs can move from one bacterial cell to another and from one bacterial species to another through horizontal gene transfer (15).

To cause bloodstream infections, *S. epidermidis* must survive in a hostile environment in the blood. As bacteria enter the bloodstream, the coagulation system usually facilitates the clearance of the invading pathogen (16). However, studies of *Staphylococcus aureus*, perhaps the most successful blood pathogen, have revealed a sophisticated mechanism to induce coagulation and coat the bacterial surface with a protective fibrinogen/fibrin shield (17). Here, we provide the first report on how invasive *S. epidermidis* ST2 strains appear to use a different strategy to accomplish a similar effect on hemostasis.

Bloodstream *S. epidermidis* ST2 isolates are shown to potentially express two CWA proteins, SesX and SesY, encoded in a putative integrated conjugative element that we call pICE-Sepi-ST2. We demonstrate that recombinant SesY interacts with human plasminogen (Plg) and plasmin (Pln) and inhibits fibrin degradation by Pln. We also discovered that the *sdrG* gene, which encodes the coagulation inhibitory CWA protein SdrG, is mutated in all isolates containing pICE-Sepi-ST2. SdrG binds to the thrombin cleavage site in the fibrinogen (Fg) beta chain and inhibits the release of fibrinopeptide B, thereby inhibiting the formation of a stable fibrin clot.

## MATERIALS AND METHODS

### Recombinant protein expression and purification

The DNA sequence encoding SesY_FL_ (residues 32–341) was cloned into a pWL613 vector (pET28b-based vector developed in-house) using In-Fusion HD Cloning Kit (Takara Bio USA) and transformed into *E. coli* Rosetta competent cells and cells were grown and induced as previously described (18). rSesY was purified using affinity chromatography (HisTrap HP column and StrepTrap HP column from Cytiva Life Sciences). Binding buffer [1.5× phosphate-buffered saline (PBS): 206 mM NaCl, 4.1 mM KCl, 15 mM Na_2_HPO_4_, 2.7 mM KH_2_PO_4_, pH 7.4, 20 mM Imidazole pH 7.5] and elution buffer (1.5× PBS, 500 mM Imidazole pH 7.5) were used for HisTrap HP affinity chromatography. Binding buffer (50 mM Tris, 250 mM NaCl pH 7.5) and elution buffer (50 mM Tris, 250 mM NaCl, 2.5 mM Desthiobiotin pH 7.5) were used for StrepTrap HP affinity chromatography. Elution from StrepTrap HP affinity chromatography was further purified by chromatography on a gel permeation column (Hi Load Superdex 200 pg 16/600 column from Cytiva Life Sciences) eluted with PBS.

### Ligand screen and solid-phase binding assay

4HBX Microtiter plate was coated with 1 µg/well of purified host proteins. Microtiter wells were blocked with 3% Bovine serum albumin (BSA) in PBS and incubated with 5 µM of recombinant proteins prepared in blocking buffer (1% BSA in PBS with 0.05% Tween-20). Recombinant proteins bound to the immobilized host ligands were detected using 1:3,000 dilution of α-His Tag HRP conjugated antibody (MAB050; R&D Systems, Minneapolis, MN) in a blocking buffer. Horseradish peroxidase (HRP) substrate *o*-phenylenediamine dihydrochloride (OPD) was added, and the resulting signal was measured at 450 nm. All incubations were at RT (room temperature) for 1 h with shaking. Wells were washed three times with PBS or PBS with 0.05% Tween-20 (PBST) after every incubation.

### Inhibition assays

Microtiter plate wells were coated with 0.5 µg/well of Plg or Pln diluted in PBS. The wells were blocked with 3% BSA in PBS. A fivefold dilution series of tranexamic acid or arginine was prepared in blocking buffer, and each dilution was added to the wells. The final concentration of tranexamic acid and arginine ranged from 0 to 10 mM. rSesY was added to the wells to a final concentration of 100 nM. The wells were washed, and rSesY bound to the host proteins was detected by incubating with α-His Tag HRP-conjugated antibody at a 1:3,000 dilution in a blocking buffer. HRP substrate ODP was added, and the resulting signal was measured at 450 nm. All the incubations were done at RT for 1 h with shaking. Wells were washed three times with PBS or PBST after every incubation.

### Pln degradation of chromogenic substrate

Fifty microliters of S-2251 (0.4 mg/mL final concentration) was mixed with 30 µL of Pln solution (1 µg/well final concentration) and 20 µL of added proteins. The total volume of all reaction components combined was 120 µL. All reagents were diluted in PBS. Digestion of S-2251 was observed at 405 nm for 1.5 h at RT using a kinetic cycle with a reading every 15 s.

### Fibrin gel degradation assay

Fibrin gel was formed by incubating Fg solution (1.25 mg/mL final concentration) and thrombin solution (0.5 NIH units/mL final concentration) in a total volume of 120 µL for 3 h at RT. Fibrin gel formed in microtiter wells was gently washed twice with 150 µL of 20 mM Tris-HCL pH 7.5. A mixture of recombinant protein (5 µM final concentration) and Pln (2 µg/well final concentration) was prepared in 100 µL total volume and added to the pre-formed fibrin gel. Degradation of fibrin was observed at 405 nm at RT using a kinetic cycle with readings every 5 min for 12 h.

### Enzyme kinetics

Enzyme kinetics was performed with rSesY, Pln, and S-2251. Different concentrations of S-2251 (3.0 × 10^−3^, 1.5 × 10^−3^, 3.6 × 10^−4^, 1.8 × 10^−4^, 9.0 × 10^−5^, and 0 M) were prepared in PBS. 10, 5, 2.5, 1, and 0 µM of rSesY and 1 µg/well final concentration Pln were added to each of the S-2251 concentrations. The kinetics of inhibition were monitored at 405 nm for 1.5 h at RT using a kinetic cycle with a reading every 15 s and plotted against the respective substrate concentration. Raw data were uploaded to ICEKAT to obtain the initial velocity (19). GraphPad Prism was used to determine the Michaelis–Menten constant (*K*_m_) and maximal velocity (*V*_max_).

### *In silico* analysis for the presence of *sesX* and *sesY* genes

Protein sequences for SesX and SesY proteins were used to query the local database containing the assembled genome sequencing data for the isolates obtained for this study. For the isolates suspected to lack *sesX* and *sesY*, the absence of these genes was confirmed by mapping the resulting reads to known *sesX* and *sesY* gene sequences.

### *In silico* analysis for the presence of the *sdrG* gene

Protein sequences of the SdrG protein were used to query the assembled genome sequencing data for the isolates obtained in this study. Coding sequences (CDS) were analyzed using Geneious Prime 2022.1.1 (https://www.geneious.com). If a CDS annotation contained an internal stop codon, Geneious Prime designated it as a premature stop codon and represented by an asterisk (*) in the translation view.

### *In silico* characterization of the pICE

The signal sequence was determined using the SignalP 5.0 server (20). Transmembrane helices in predicted proteins were identified using the TMHMM 2.0 server (21). Conserved domains in the proteins were identified using the Batch Web CD-Search tool (22) and Phyre2 (23). The sub-cellular location of proteins was determined using PSORTb 3.0 (24). All analyses use the default parameters, ensuring consistency and comparability of the results.

*In silico* structure prediction SesX and SesY’s full-length structures were predicted using AlphaFold 2.1.1 (25) in the monomer mode. SesX and SesY prediction models were displayed using ChimeraX (26).

### Statistical analysis

To compare multiple groups, we used a one-way ANOVA followed by Dunnett’s multiple-comparison tests. The *P*-values, representing the comparisons between groups, are reported as follows: *P < 0.0118 or <0.0142, **P < 0.0067, and ****P < 0.0001 or <0.000001. All statistical analyses were performed using GraphPad Prism software 10.0.3.

## RESULTS

### Genome comparison of *S. epidermidis* bloodstream isolates

**Fig 1:**
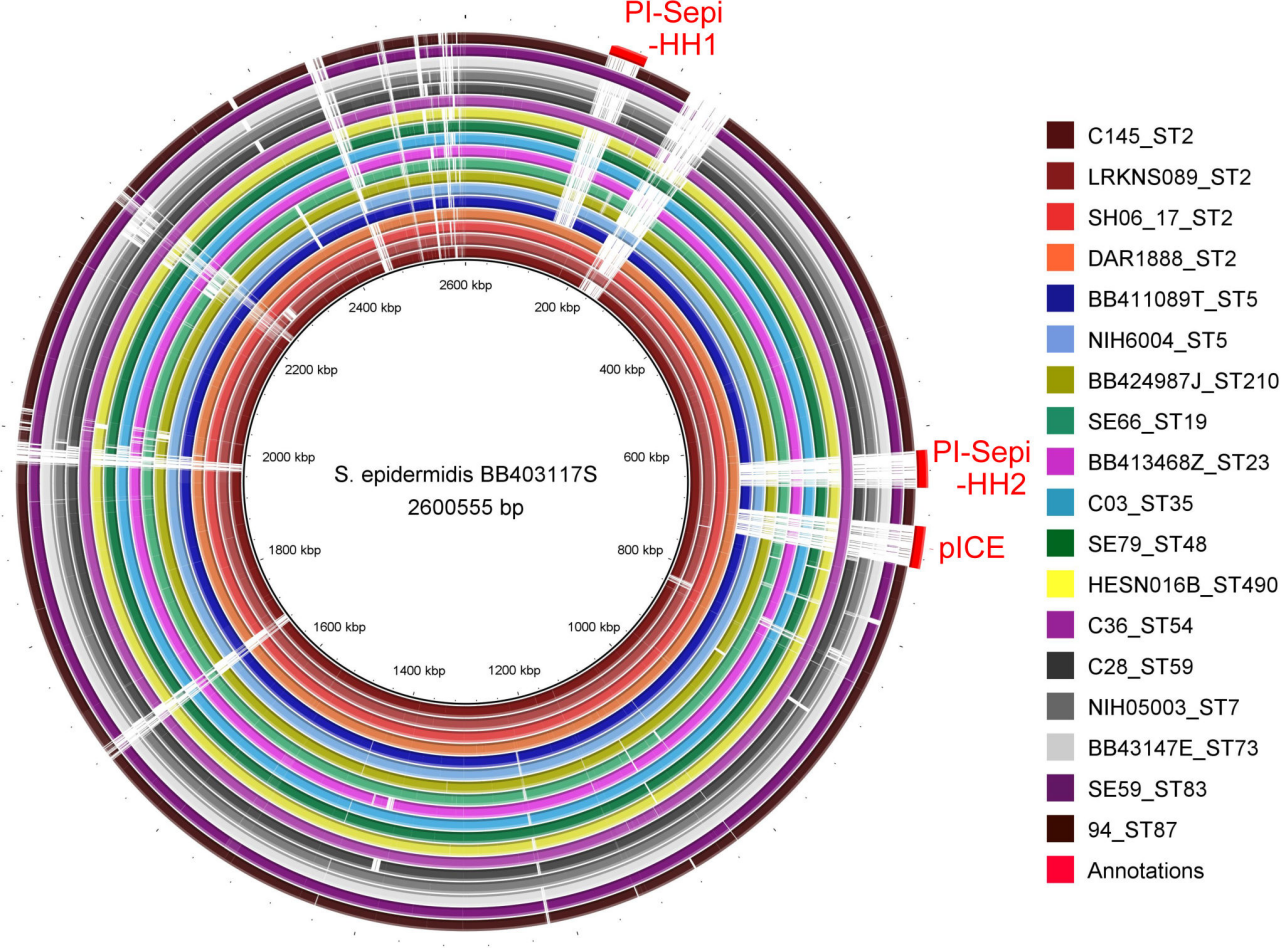
Genome comparison of global *S. epidermidis* isolates obtained from human blood. The circular genome of ST2 *S. epidermidis* BB403117S was compared using BLAST to 18 other *S. epidermidis* isolates from 14 different sequence types (STs). The legend of the comparison map includes the name of each *S. epidermidis* isolate followed by its sequence type (format: Strain_ST). Annotations around the outer ring highlight genomic regions present in ST2 bloodstream isolates but absent in other STs, except for ST54. The genomic data for these isolates were sourced from BV-BRC, and the circular genome comparative map was generated using BRIG (30). For more detailed information on the isolates, see Table S1.

To uncover why specifically ST2 isolates are commonly associated with bloodstream infections, we focused on identifying over-represented MGEs in *S. epidermidis* ST2 bloodstream isolates. To this end, we identified global *S. epidermidis* bloodstream isolates across different STs with deposited genome assemblies in the Bacterial and Viral Bioinformatics Resource Center (BV-BRC) (27–29). We compared the genomes of ST2 isolates with those of other STs (Fig. 1; Table S1). We restricted our analysis to random isolates obtained from human blood with a known ST and were annotated to have a high-quality genome sequence by BV-BRC. *S. epidermidis* BB403117S ST2 genome was compared to 4 ST2 isolates and 14 other isolates (2 ST5, 1 isolate each of ST210, ST19, ST23, ST35, ST48, ST490, ST54, ST59, ST7, ST3, ST83, and ST87). From this initial experiment, we identified three regions present in all four ST2 isolates and notably absent in other STs except for ST54. ST54 is a closely related variant of ST2 and differs from ST2 by only one allele. Further investigation of the regions revealed these elements to be (i) ɸSepi-HH1, a complete phage (Accession number: MT880870), (ii) PI-Sepi-HH2, a phage-related island (Accession number: MT880871), and (iii) a putative Integrated Conjugative Element (pICE) named here as pICE-Sepi-ST2 (Accession number: OP831990, OP831991, OP831992, OP831993, OP831994).

### Characterization of the putative integrated conjugative element Sepi-ST2

Next, we searched for open reading frames that potentially encode CWA proteins in the unique ST2 MGEs since CWA proteins often act as virulence factors in Gram-positive pathogens. Among ɸSepi-HH1, PI-Sepi-HH2, and pICE-Sepi-ST2, only the pICE-Sepi-ST2 encodes putative CWA proteins; two were identified that we have named *S. epidermidis* surface protein X (SesX) and Y (SesY). We focused on characterizing the pICE-Sepi-ST2 and the CWA proteins SesX and SesY.

**Fig 2:**
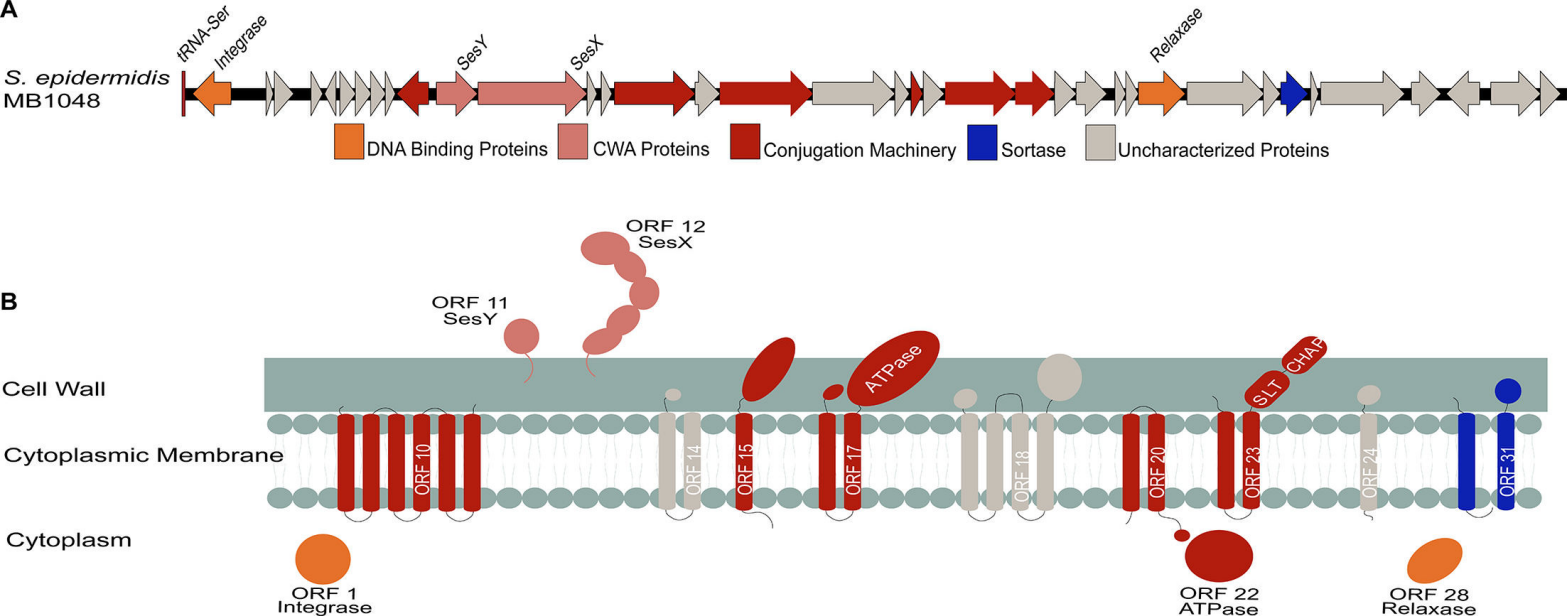
Schematic of pICE-ST2 found in *S. epidermidis* ST2 bloodstream isolates**.** (**A**) Genetic organization of the open reading frame in pICE-ST2. Genes are drawn to scale. Genes are colored based on their function: DNA-binding proteins are shown in orange; CWA proteins are shown in pink; genes for conjugation machinery are shown in red; *sortase* gene is colored blue; genes with unknown functions are colored gray. (**B**) Domain organization, location, and topologies of proteins with known predicted functions are shown. Proteins are colored as in panel A based on their predicted gene functions.

The pICE-Sepi-ST2 meets the criteria for a pICE since it encodes for an integrase, a relaxase, and a VirB4-like protein (31). The pICE-Sepi-ST2 is integrated into the 3′ end of the tRNA^Ser GGA^ gene in ST2 isolates and is 38,102 bp in length (Fig. 2A). The encoded ORFs can be divided into different families, including conjugation machinery, DNA binding proteins, and CWA proteins (Fig. 2A and B; Supplemental results). Multiple uncharacterized proteins and a sortase enzyme are encoded in the pICE (Fig. 2). We also compared the structure of pICE in MB1048 with that of the other *S. epidermidis* ST2 bloodstream isolates. We found that the overall organization of the ORFs in pICE is conserved across the isolates analyzed (Fig. S1).

### pICE-Sepi-ST2 is present in all *S. epidermidis* ST2 bacteremia isolates analyzed

We examined the prevalence of pICE-Sepi-ST2 in a collection of 171 *S. epidermidis* bacteremia isolates obtained in the USA (11, 32). Using *sesX* and *sesY* genes as markers for pICE-Sepi-ST2 in an *in-silico* analysis, we determined that the *sesX* and *sesY* genes and subsequently pICE-Sepi-ST2 were present in 34/34 ST2 blood isolates in this collection (Table 1; Table S2). We found that the *sesX* and *sesY* genes were specific to ST2 isolates in this collection and were not present in other *S. epidermidis* STs (Table 1; and Table S2).

**TABLE 1:** Presence of *sesX* and *sesY* genes and *sdrG* gene truncation in *S. epidermidis* isolates from blood

ST	No. of isolates	*sesX^+^* isolates *n* (% of ST)	s*esY^+^* isolates *n* (% of ST)	*sdrG* truncation *n* (% of ST)
ST 2	34	34 (100)	34 (100)	34 (100)
ST 83	17	0	0	0
ST 210	6	0	0	0
ST 5	59	0	0	0
ST 16	5	0	0	1 (20)
ST 20	3	0	0	2 (67)
ST22	5	0	0	0
ST59	4	0	0	0
ST69	3	0	0	0
ST130	3	0	0	0
ST6	4	0	0	0
Rare	29	0	0	10 (21)
*Total*	*171*	*34 (19.8)*	*34 (19.8)*	*47 (27)*

To examine the presence of the *sesX* and *sesY* genes in different types of infections caused by ST2 strains, we used a collection of publicly available genomes of ST2 isolates from different infection sources. We identified a total of 77 ST2 *S. epidermidis* isolates that were present in the BV-BRC database and were collected in Germany from catheter, fracture fixation, prosthetic joint infection, and bacteremia, respectively (Table 2; Table S3). We observed that the *sesX* and *sesY* genes are present in all the isolates from bloodstream infection (100%) but were only present in a portion of the isolates obtained from the catheter (44%), fracture fixation (27%), and prosthetic joint (52%) infections (Table 2; Table S3).

**TABLE 2:** Presence of *sesX* and *sesY* genes and *sdrG* gene truncation in *S. epidermidis* Germany ST2 isolates from different sources

Isolation source	No. of isolates	*sesX^+^* isolates *n* (%)	*sesY^+^* isolates *n* (%)	*sdrG* truncated isolates *n* (%)
Catheter	27	12 (44)	12 (44)	14 (52)
Fracture fixation	11	3 (27)	3 (27)	10 (91)
Prosthetic joint infection	29	17 (52)	17 (52)	20 (69)
Bacteremia	10	10 (100)	10 (100)	10 (100)
*Total*	*77*	*42 (55)*	*42 (55)*	*54 (70)*

Combined, our genome analysis demonstrates that the sesX and sesY genes are only present in ST2 *S. epidermidis* strains and found in all ST2 *S. epidermidis* bacteremia isolates examined and present only in some ST2 isolates obtained from infection sites other than blood (Tables 1 and 2).

### The putative CWA proteins, SesX and SesY

**Fig 3:**
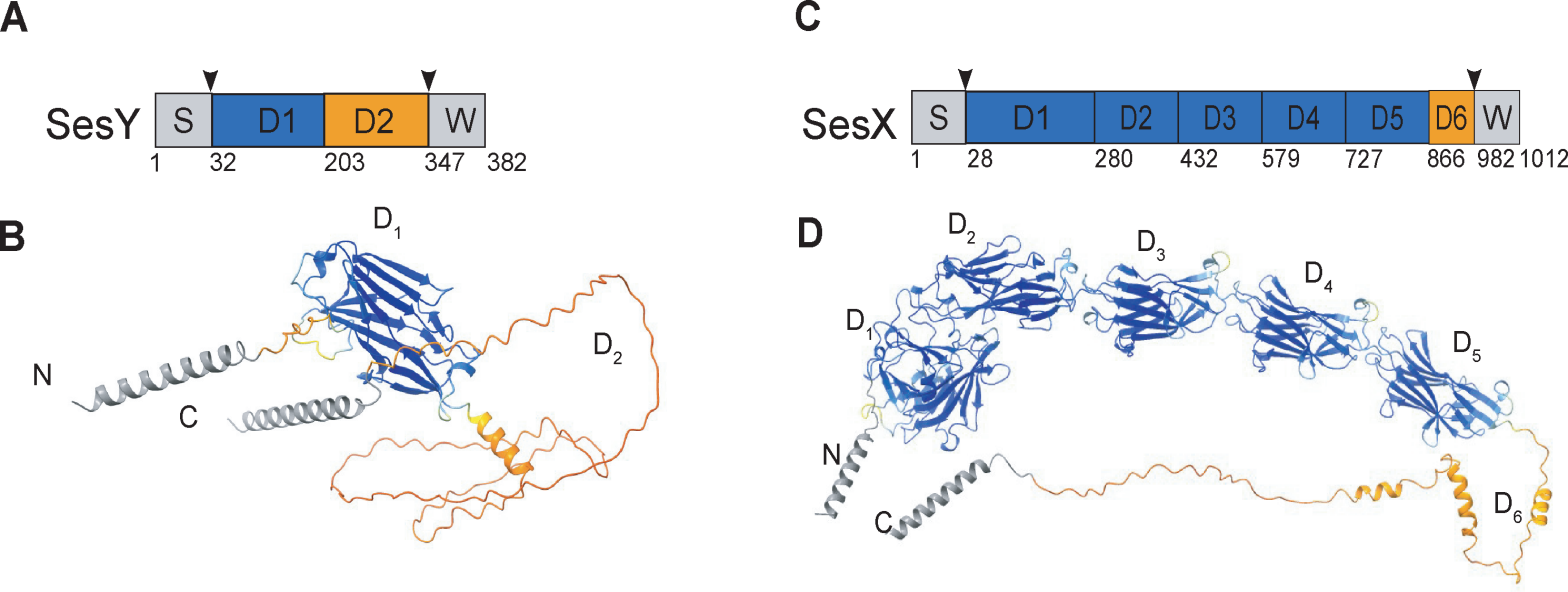
Predicted domain organizations and structures of SesY and SesX proteins. (**A and C**) The predicted domain organization of SesY and SesX is illustrated in panels (**A**) and (**C**), respectively. The signal sequence (S) is shown in gray. Following the signal sequences, SesY and SesX have multiple domains labeled numerically as D_1_ to D_*n*_. Inverted black arrows indicate the "full-length" protein (SesY_32–341_ and SesX_28–982_). The cell wall, membrane-spanning region, and short cytoplasmic tail are collectively labeled as W, as shown in gray. The numbers below the schematic indicate the start of each domain, except for the last number, which represents the total number of amino acids in the protein. D-domains are color-coded based on the Alphafold 2 LDDT (per residue confidence metric) score: blue indicates a score over 70, while orange indicates a disordered region with a score below 50. (**B and D**) Predicted structure of SesY and SesX through Alphafold 2. N- and C-terminus are indicated. Amino acids in the domains are colored based on the LDDT scores.

Next, we analyzed models of the putative ST2 blood strain-associated CWA proteins, SesX and SesY, which were predicted using AlphaFold2 (25) (Fig. 3). The deduced full-length SesX and SesY proteins in ST2 *S. epidermidis* MB1048 are 1012 a.a. and 382 a.a. long, respectively. SesX and SesY are multidomain proteins containing the typical signal sequences at the N-terminus. At the C-terminal regions, we identified the characteristic motifs for CWA proteins, including an LPXTG sequence, cell wall, membrane-spanning areas, and short positively charged cytoplasmic tail (33) (Fig. 3A and C). Between the N-terminus signal sequence and the LPXTG motif models, the two proteins contain multiple domains assigned sequentially as D_*n*_, where *n* is the number of the domain (Fig. 3). The SesY protein model contains two D-domains, D_1_ and D_2_, where the D_1_domain has a folded structure that contains mostly β-sheets and three small α-helices. The D_2_ domain is predicted to contain two short alpha helices, but most of the domain is scored as disordered, where all residues have <50 pLDDT (per residue confidence metric) values (Fig. 3B). The SesX protein model includes six D-domains, where D_1–5_ domains are arranged head to tail to form an extended structure, with the D_1_ domain forming the head of the protein. The D6 domain is predicted to be disordered, and residues have <50 pLDDT values. D_1–5_ have a similar secondary structure composition with mostly beta sheets and 2–3 small alpha-helices interspersed within each domain (Fig. 3D).

**Fig 4:**
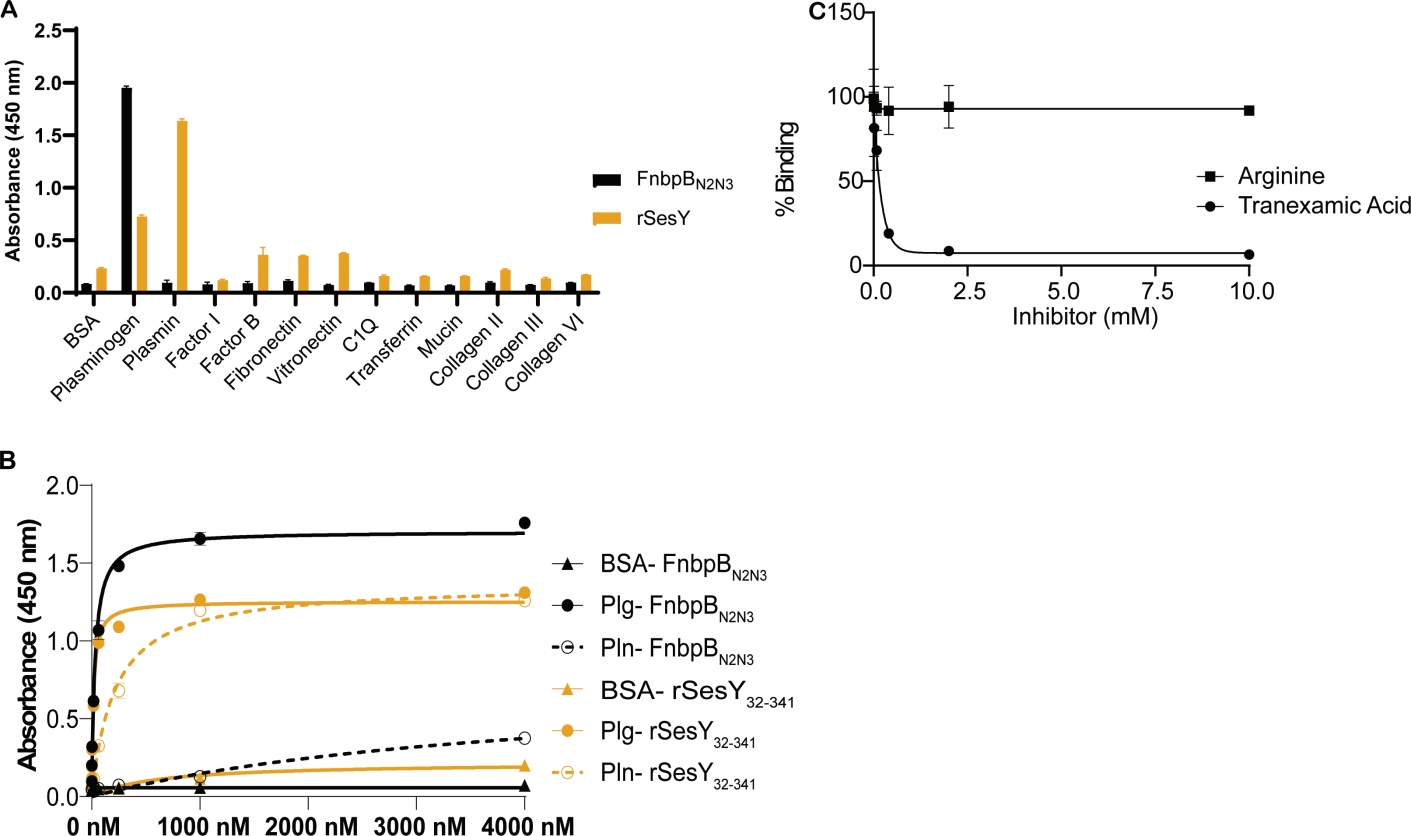
Binding of SesY to Plg and Pln. (**A**) Host ligands were immobilized on 4HBX microtiter plates to evaluate for binding of His-tagged recombinant proteins rSesY and FnbPB_N2N3_. Detection was performed using an anti-His antibody. Bovine serum albumin (BSA) served as a negative control. (**B**) Different concentrations of recombinant proteins rSesY and FnbPB_N2N3_ (positive control) were evaluated for binding to immobilized human Plg and Pln on 4HBX microtiter plates. Binding was detected in a dose-dependent manner using α-His Tag HRP conjugated antibody (1:3,000). The binding curve and dissociation constant were approximated via non-linear regression using a one-site, specific binding model. (**C**) Inhibition of rSesY binding to immobilized Pln was assessed in varying concentrations of a lysine derivative, tranexamic acid (circle), and arginine (square). rSesY bound to Pln was detected with α-His Tag HRP conjugated antibody at a 1:3,000 dilution. The presence of tranexamic acid decreased rSesY binds to Pln by 85%. Results from three replicates (*n* = 3) are represented. Error bars indicate the standard deviation (mean ± SD).

We focused on a possible ligand for SesY and conducted a ligand screen with known host protein targets of Gram-positive CWA proteins (34–36). The host proteins were immobilized in microtiter wells and incubated with 1 µM of recombinant SesY_32–341_ (rSesY). rSesY represents a full-length mature SesY protein that excludes the C-terminal cell wall anchoring domain. To facilitate protein purification, we added a N-terminus His-tag and a C-terminus Strep-tag. Α-His HRP antibody was used to detect the binding of rSesY to host proteins. In this assay, rSesY bound both Plg and Pln. No binding was observed for immobilized factor I, factor B, fibronectin, transferrin, mucin, vitronectin, laminin, C1q, collagen II, collagen III, and collagen VI (Fig. 4A). Fibronectin-binding protein B (FnbpB) from *S. aureus* has also been shown to bind fibrinogen (Fg) and Plg (37). The minimal binding regions for Fg and Plg in FnbpB are in the N_2_N_3_ domain (37). Therefore, rFnbpB_N2N3_ was used as a positive control and, as expected, bound to human Plg (Fig. 4A) but not to Pln (37).

### Characterization of SesY’s interaction with human Plg and Pln

The binding of SesY to Plg and Pln was further characterized in solid-phase binding assays. Full-length Plg and Pln were immobilized and were incubated with increasing concentrations (1 nM to 4 µM) of rSesY. The rSesY bound to immobilized Plg and Pln in a dose-dependent and saturable manner with an apparent *K*_D_ of 2.1 × 10 ^−7^ M and 1.6 × 10 ^−8^ M, respectively (Fig. 4B). rFnbpB_N2N3_ protein was used as a positive control for Plg binding and showed an apparent KD of 3.0 × 1 0 ^−8^ M (Fig. 4B). Again, the rFnbpB_N2N3_ did not bind to Pln with comparable affinity. Bovine serum albumin (BSA) was used as a negative control, and rSesY and rFnbpB_N2N3_ did not show binding to BSA (Fig. 4B).

The Kringle domains of Plg/Pln contain “lysine binding sites” and bind to lysine residues in the interacting proteins. To further characterize the interaction of SesY with Plg and Pln, we tested if the Plg and Pln bind to the lysine residues present in rSesY. Plg or Pln coated in microtiter wells was incubated with a fixed concentration of rSesY and an increasing concentration of tranexamic acid. This clinically approved Plg activation inhibitor blocks the binding of Plg/Pln to lysine residues in their ligands (38). Our results show that the binding of rSesY to both Plg and Pln decreased with increasing concentrations of tranexamic acid (Fig. 4C; Fig. S2). Tranexamic acid at 2 mM reduced the binding of rSesY to Plg and Pln by over 85% (Fig. 4C; Fig. S2), indicating that both Pln and Plg bind to the lysine residues present in the SesY protein. Inhibition of SesY and Plg/Pln binding was specific to tranexamic acid, as arginine, another positively charged amino acid, did not inhibit the binding of rSesY to Plg/Pln (Fig. 4C; Fig. S2).

### SesY inhibits Pln activity

**Fig 5:**
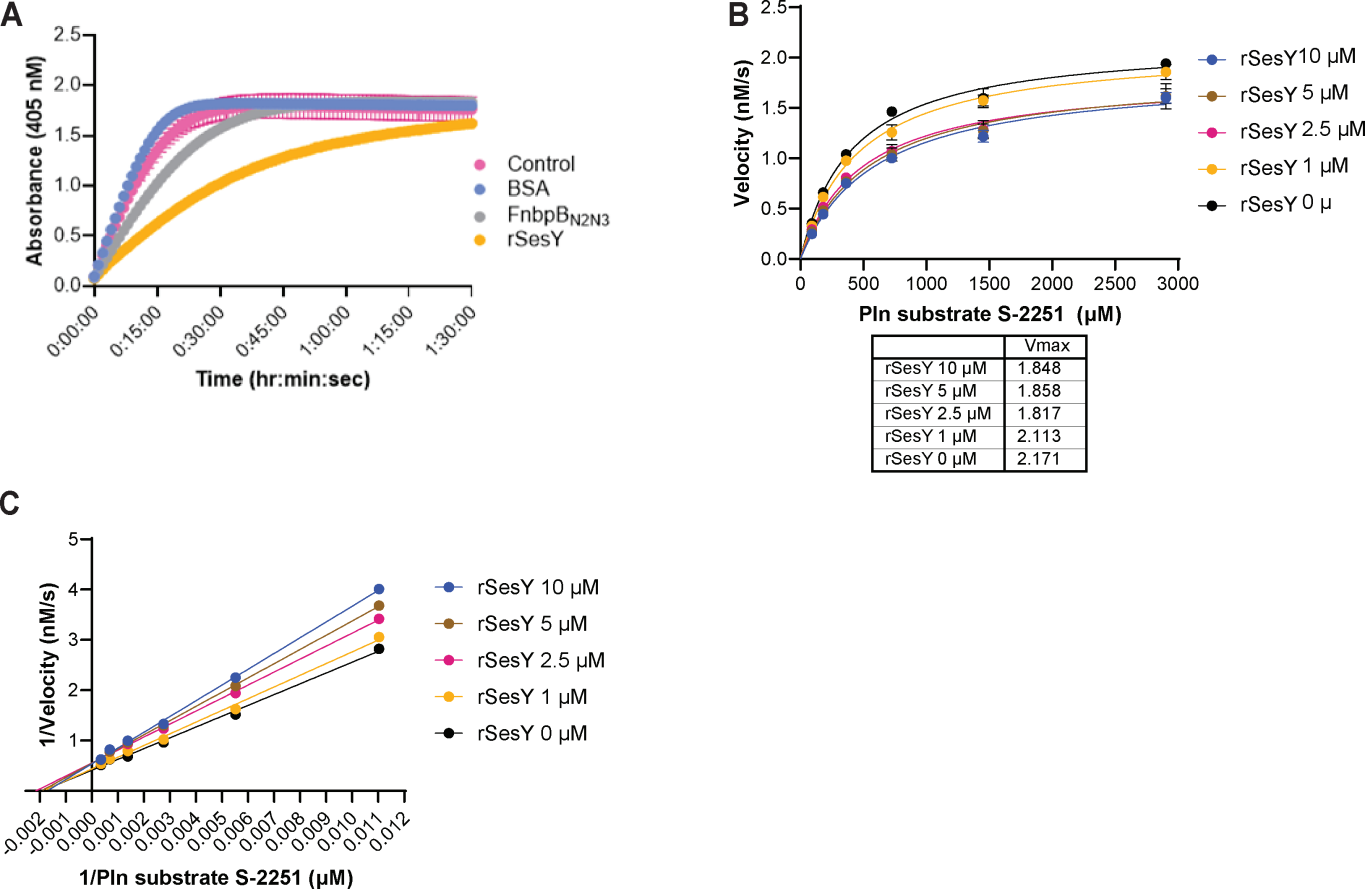
SesY inhibits Pln activity. (**A**) Graph representing color development from chromogenic Pln substrate S-2251 digestion at different time points during 1.5 h in the presence of 5 µM of rFnbpB_N2N3_, rSesY, and BSA. Control represents wells with no additional protein added to the reaction. (**B**) The initial velocities of Pln are plotted against various concentrations of the Pln S-2251 substrate to generate Michaelis-Menten curves. This was done in the presence of increasing concentrations of the recombinant protein rSesY (0 µM, 1 µM, 2.5 µM, 5 µM, 10 µM) to evaluate its effect on Pln activity. (**C**) Lineweaver-Burk plots are generated from Michaelis-Menten curves to analyze the enzyme (Pln)-substrate (S-2251)-inhibitor (rSesY) relationship. Noncompetitive inhibition is shown in the presence of rSesY since *K*_m_ is unaffected and *V*_max_ is reduced. Results from three replicates (*n* = 3) are represented. Error bars indicate the standard deviation (mean ± SD).

To determine if the binding of SesY to Pln influences Pln activity, we analyzed the proteolytic activity of Pln in the presence and absence of rSesY using the chromogenic substrate D-Valyl-l-leucyl-l-lysine 4-nitroanilide dihydrochloride [S-2251 (39)]. We found that rSesY inhibited cleavage of the chromogenic substrate S-2251 by Pln, whereas BSA and control did not (Fig. 5A). To further characterize the inhibiting effect of SesY, we determined Pln kinetics in the presence of different concentrations of rSesY protein. We observed a significant decrease in *V*_max_ for the Pln and S-2251 from 2.1 nM/min to 1.8 nM/min in the presence of rSesY (Fig. S3A). The increase in the *K*_m_ for Pln and S-2251 interaction was not statistically significant (Fig. 5B and C; Fig. S3B). Combined, our data suggest that (i) the inhibitory effect of SesY on Pln activity is due to a direct interaction between SesY and Pln and (ii) SesY acts as a non-competitive inhibitor of Pln’s proteolytic activity.

**Fig 6:**
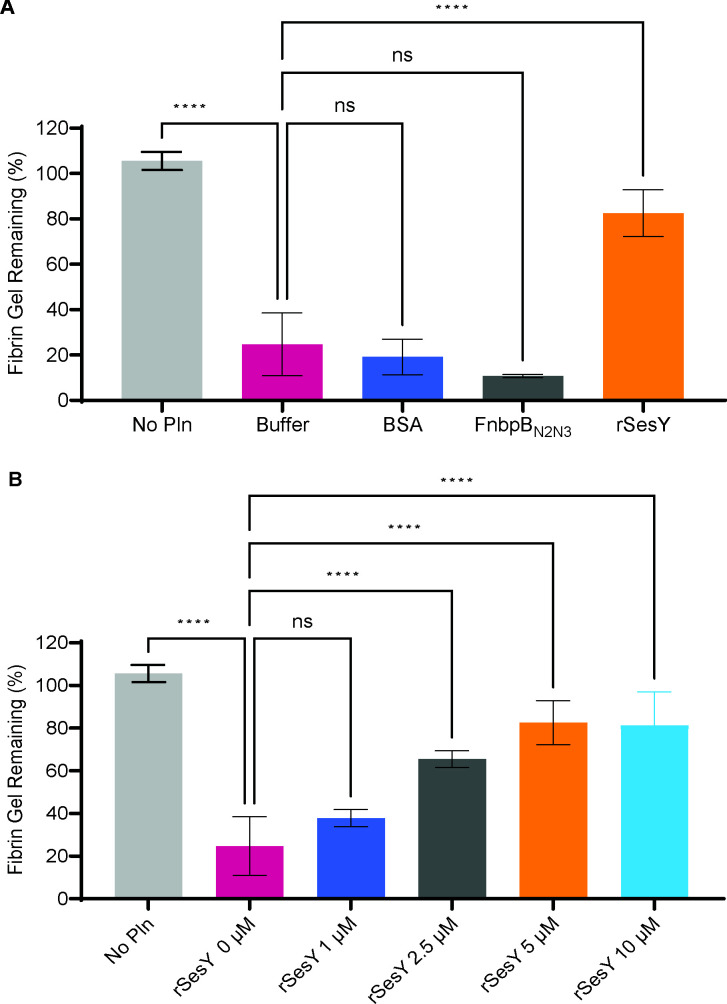
SesY inhibits the Pln-mediated degradation of fibrin**.** (**A**) Percentage of fibrin gel remaining after digestion of pre-formed fibrin gel at 12 h in the presence of Pln and 5 µM of different proteins: rFnbpB_N2N3_, rSesY, and BSA. Buffer control represents wells with 0 µM of additional protein. No Pln represents wells with pre-formed fibrin gel without the addition of Pln. (**B**) Percentage of fibrin gel remaining after digestion of pre-formed fibrin gel at 12 h time point in the presence of Pln and different concentrations of rSesY (0 µM, 1 µM, 2.5 µM, 5 µM, 10 µM). Buffer control represents wells with 0 µM of additional protein. No Pln represents wells with pre-formed fibrin gel without the addition of Pln. Results from three replicates *n* = 3 is represented. Error bars indicate the standard deviation. One-way ANOVA followed by Dunnett’s multiple-comparison tests (mean ± SD, ****P < 0.000001 [**A**] and *P* < 0.00001 [**B**]).

Pln’s major physiological function is in fibrinolysis, where it degrades fibrin to fibrin-degradation products (40). Therefore, we tested whether SesY inhibits Pln degradation of fibrin, its major natural substrate. To this end, we developed a fibrin degradation assay where preformed fibrin gels were incubated with Pln in the presence or absence of rSesY at a final concentration of 5 µM. Fibrin gels were formed in the microtiter wells by incubating purified Fg with thrombin for 3 h. Subsequently, rSesY, rFnbpB_N2N3_, and Pln were added. rSesY inhibited fibrin gel degradation by Pln, whereas rFnbpB_N2N3_ had no effect (Fig. 6A; Fig. S4A). At a 12 h timepoint, wells containing 5 µM rSesY had 80% of the fibrin gel remaining compared to controls containing buffer or rFnbpB_N2N3_, which had 10%–25% fibrin remaining. Next, we tested the effect of different concentrations of rSesY on the degradation of preformed fibrin gel by Pln. We observed a clear dose-dependent response, where 5 and 10 µM exhibited the highest response, with over 80% of the fibrin gel remaining at both the 6 and 12 h timepoints (Fig. 6B; Fig. S4B). Lower concentrations of rSesY (2.5 and 1 µM) also showed inhibitory effects, albeit to a lesser extent, with 79% and 65% remaining fibrin gel with 2.5 µM and 59% and 38% remaining fibrin gel with 1 µM at 6 h and 12 h, respectively (Fig. 6B; Fig. S4B).

### The *sdrG* gene is mutated in ST2 *S. epidermidis* isolates

The core genomes of most *S. epidermidis* strains, including ST2s, contain the gene for the CWA protein SdrG. Our group has previously shown that SdrG binds to Fg at the N-terminus of the β-chain (41) and inhibits the thrombin-catalyzed release of fibrinopeptide B (41), thus interfering with the formation of the blood clot. This would appear to have the opposite effect on coagulation hemostasis compared to SesY, which we now show inhibits fibrinolysis. Therefore, we decided to look at the *sdrG* gene in the clinical isolates examined in this study. We discovered that in ST2 *S. epidermidis* bacteremia isolates from the US collection, the *sdrG* gene had a stop codon in the coding sequence, leading to premature translation termination in all 34 isolates (Table 1; Table S2). The *sdrG* gene with a stop codon in the coding sequence was also identified in ST16, ST20, and ST10 rare isolates belonging to STs with two or fewer isolates in the collection.

Similarly, in the ST2 Germany isolates from different infection sources, the *sdrG* gene had a stop codon in the coding sequence in 54 out of 77 isolates (Table 2; Table S3). All the ST2 bloodstream Germany isolates had a mutated *sdrG* gene. In contrast, *sdrG* mutation in ST2 isolates from other types of infections was less frequent: fracture fixation (91%), the catheter (52%), and prosthetic joint infection (69%) (Table 2).

**Fig 7:**
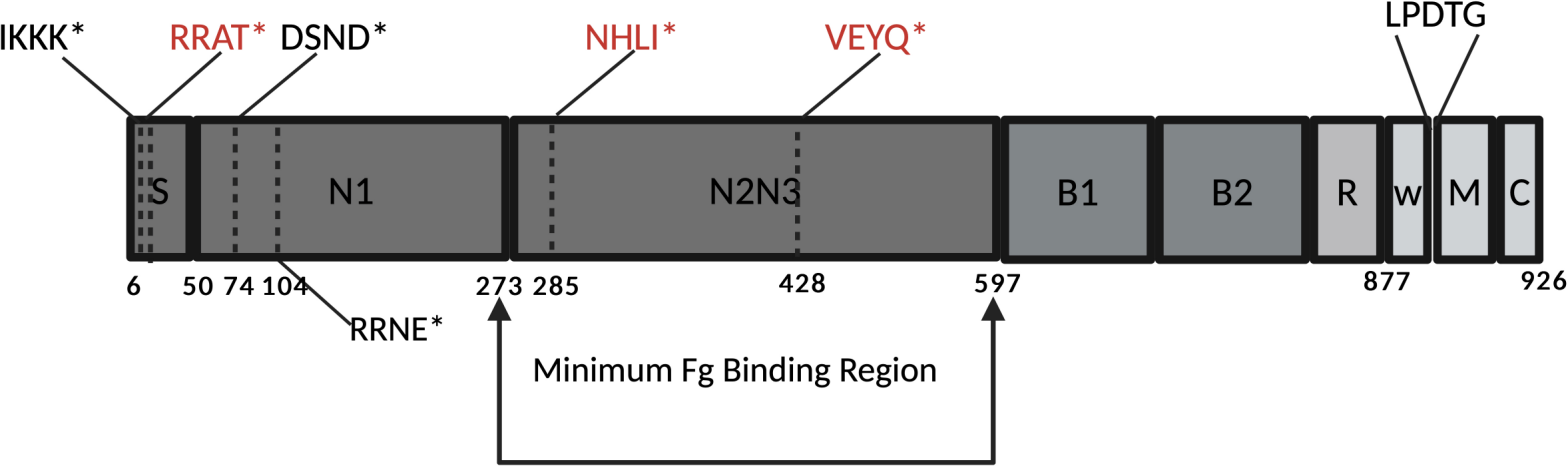
Schematic of truncations in the SdrG protein found in *S. epidermidis* ST2 isolates. The figure illustrates the different domains of the SdrG protein: the signal sequence is labeled S; the N-terminal domains are labeled N1, N2, and N3; B1 and B2 are repeats of unknown function; R stands for the serine-aspartate repeat region; W stands for the cell wall region; and M for the membrane-spanning region. The dotted line indicates the position of the stop codon. The figure shows the amino acid sequence followed by an asterisk (*) to represent the truncation of the protein at that position. Stop codons found in ST2 bloodstream isolates are highlighted in red.

It is noteworthy that the *sdrG* gene in all *sesY*-positive ST2 isolates was mutated, leading to premature translation termination (Fig. 7; Table S2). However, not all isolates with a mutated *sdrG* gene contained a *sesY* gene. Of the ST2 isolate, all but one harbored a stop codon after VEVQ in the minimum Fg binding region of SdrG (Fig. 7). Other non-ST2 isolates with mutated *sdrG* genes contain different stop codons along the gene (Fig. 7).

## DISCUSSION

*S. epidermidis*, earlier viewed as a benign commensal bacterium, has recently gained recognition as an opportunistic pathogen, particularly in nosocomial and device-related infections. This bacterium’s genetic heterogeneity, characterized by numerous STs, is likely responsible for its adaptability and pathogenicity in different environments. ST2 has been identified in invasive *S. epidermidis* infections worldwide (1–3, 6). However, phylogenetic analyses of *S. epidermidis* core genomes fail to differentiate between commensal and infectious isolates (3, 6). We now report on an MGE, pICE-Sepi-ST2, encoding two CWA proteins, SesX and SesY, found exclusively in ST2 strains. More importantly, pICE-Sepi-ST2 is present in all ST2 bloodstream isolates but only in portions of isolates from other infection sources. This observation strongly indicates an association between this MGE and the ability of *S. epidermidis* ST2 to cause bloodstream infections. The pICE-Sepi-ST2, and possibly the *sesX* and *sesY* genes it carries, could be important virulence factors in the context of bloodstream infections. Furthermore, our comparative genome analysis of bloodstream isolates highlights the absence of *sesX* and *sesY* genes in other STs, suggesting the existence of other virulence mechanisms or adaptive strategies that cause bloodstream infections in other STs. Taken together, our findings provide a direct link between pICE-Sepi-ST2 and the invasiveness of ST2 *S. epidermidis* isolates and highlight the role of MGEs in increasing the virulence potential of harboring *S. epidermidis* strains.

Most *S. epidermidis* strains encode another CWA protein in the core genome, SdrG, which manipulates the host’s hemostatic system. SdrG binds to the thrombin cleavage site in the Beta chain of Fg, inhibiting the thrombin release of fibrinopeptide B and, hence, the fibrin formation (41). Thus, SdrG inhibits fibrin formation, and SesY inhibits fibrin degradation, two actions that have apparent contradicting functions in hemostasis. However, analysis of the *sdrG* gene in ST2 bloodstream isolates revealed the presence of a stop codon that would prevent the translation of active SdrG protein. The inactivation of the *sdrG* gene was seen in all ST2 bloodstream isolates analyzed but only in some of the isolates obtained from other types of infections. ST54 being a close relative of ST2 also had a premature stop codon in the Fg-binding region. The consistent presence of two genomic modifications—namely, the MGE pICE-Sepi-ST2 encoding SesY and the inactivation of SdrG—across all ST2 bloodstream isolates strongly suggests a purposeful adaptation strategy directed at the hemostasis pathway.

For this study, we focused our attention on SesY. We demonstrate that SesY binds to Plg and its active form, Pln, and inhibits Pln’s activity. Our data suggest that SesY is a noncompetitive inhibitor of Pln and potentially binds to “lysine binding sites” of Plg/Pln Kringle domains (42–44). The complex role of Pln in host-pathogen interactions has been extensively studied (42, 43, 45, 46). As a central player in the host fibrinolytic system, Pln regulates blood fluidity by degrading fibrin and modulates inflammation and immune responses. Its role in activating matrix metalloproteinases, cytokines, chemokines, and processing complement and kinin system components, aiding in modulating these critical physiological responders (47, 48). A study by Guo et al. (43) demonstrates the dichotomous role of Pln during infection and sepsis (43). They found that in systemic *S. aureus* infection, Plg-deficient mice displayed lower survival rates compared to wild-type mice, underscoring Pln’s protective role (43). In contrast, during sepsis, mice with high levels of active Pln showed the lowest survival rates implicating active Pln exacerbating host defense and enhancing bacterial survival (43). By impeding Pln, SesY could help *S. epidermidis* evade the host’s defense mechanism in the bloodstream, where Pln activity appears critical (43, 49).

This report sheds light on the multifaceted nature of *S. epidermidis* pathogenesis, revealing a diversity of adaptation strategies specific to different STs. In conclusion, our study provides novel insights into the distinct genomic modification of *S. epidermidis* ST2 bloodstream isolates, unveiling a potentially important role for the MGE pICE-Sepi-ST2 and one of its encoded proteins, SesY, in virulence.

## Data Availability

The genomic data supporting the findings of this study have been deposited in the National Center for Biotechnology Information (NCBI). Detailed information, including all BioProjects, is provided in the supplemental material. Table S1 contains the BioProjects for the global *S. epidermidis* blood isolates from BV-BRC, and Table S2 contains the BioProjects for the Germany and MDA isolates. All data are publicly accessible at NCBI (https://www.ncbi.nlm.nih.gov/) and BV-BRC (https://www.bv-brc.org). The AlphaFold PDB files for SesX and SesY are available by request via email to the corresponding author.

## SUPPLEMENTAL MATERIAL

Supplemental material mbio.01907-24-s0001.docx Supplemental text and figures.

Table S1 mbio.01907-24-s0002.xlsx Information on BV-BRC isolates from Fig. 1.

Tables S2 and S3 mbio.01907-24-s0003.xlsx Information on SrdG mutation for MDA and German isolates.

ASM does not own the copyrights to Supplemental Material that may be linked to, or accessed through, an article. The authors have granted ASM a non-exclusive, world-wide license to publish the Supplemental Material files. Please contact the corresponding author directly for reuse.

## References

[1] Deplano A, Vandendriessche S, Nonhoff C, Dodémont M, Roisin S, Denis O. 2016. National surveillance of Staphylococcus epidermidis recovered from bloodstream infections in Belgian hospitals. J Antimicrob Chemother 71:1815–1819. doi:10.1093/jac/dkw086 27118780

[2] Lee JYH, Monk IR, Gonçalves da Silva A, Seemann T, Chua KYL, Kearns A, Hill R, Woodford N, Bartels MD, Strommenger B, Laurent F, Dodémont M, Deplano A, Patel R, Larsen AR, Korman TM, Stinear TP, Howden BP. 2018. Global spread of three multidrug-resistant lineages of Staphylococcus epidermidis. Nat Microbiol 3:1175–1185. doi:10.1038/s41564-018-0230-7 30177740 PMC6660648

[3] Shelburne SA, Dib RW, Endres BT, Reitzel R, Li X, Kalia A, Sahasrabhojane P, Chaftari A-M, Hachem R, Vargas-Cruz NS, Jiang Y, Garey K, Fowler VG Jr, Holland TL, Gu J, Miller W, Sakurai A, Arias CA, Aitken SL, Greenberg DE, Kim J, Flores AR, Raad I. 2020. Whole-genome sequencing of Staphylococcus epidermidis bloodstream isolates from a prospective clinical trial reveals that complicated bacteraemia is caused by a limited number of closely related sequence types. Clin Microbiol Infect 26:646. doi:10.1016/j.cmi.2019.10.008 31639470

[4] Widerström M, McCullough CA, Coombs GW, Monsen T, Christiansen KJ. 2012. A multidrug-resistant Staphylococcus epidermidis clone (ST2) is an ongoing cause of hospital-acquired infection in a Western Australian hospital. J Clin Microbiol 50:2147–2151. doi:10.1128/JCM.06456-11 22442320 PMC3372155

[5] Mekni MA, Bouchami O, Achour W, Ben Hassen A. 2012. Strong biofilm production but not adhesion virulence factors can discriminate between invasive and commensal Staphylococcus epidermidis strains. APMIS 120:605–611. doi:10.1111/j.1600-0463.2012.02877.x 22779682

[6] Méric G, Mageiros L, Pensar J, Laabei M, Yahara K, Pascoe B, Kittiwan N, Tadee P, Post V, Lamble S, Bowden R, Bray JE, Morgenstern M, Jolley KA, Maiden MCJ, Feil EJ, Didelot X, Miragaia M, de Lencastre H, Moriarty TF, Rohde H, Massey R, Mack D, Corander J, Sheppard SK. 2018. Disease-associated genotypes of the commensal skin bacterium Staphylococcus epidermidis. Nat Commun 9:5034. doi:10.1038/s41467-018-07368-7 30487573 PMC6261936

[7] Qi X, Jin Y, Duan J, Hao Z, Wang S, Guo Y, Lv J, Hu L, Wang L, Yu F. 2017. SesI may be associated with the invasiveness of Staphylococcus epidermidis Front Microbiol 8:2574. doi:10.3389/fmicb.2017.02574 29354100 PMC5758504

[8] Salgueiro VC, Iorio NLP, Ferreira MC, Chamon RC, dos Santos KRN. 2017. Methicillin resistance and virulence genes in invasive and nasal Staphylococcus epidermidis isolates from neonates. BMC Microbiol 17:15. doi:10.1186/s12866-017-0930-9 28086793 PMC5237318

[9] Yang X-M, Li N, Chen J-M, Ou Y-Z, Jin H, Lu H-J, Zhu Y-L, Qin Z-Q, Qu D, Yang P-Y. 2006. Comparative proteomic analysis between the invasive and commensal strains of Staphylococcus epidermidis. FEMS Microbiol Lett 261:32–40. doi:10.1111/j.1574-6968.2006.00327.x 16842355

[10] Frost LS, Leplae R, Summers AO, Toussaint A. 2005. Mobile genetic elements: the agents of open source evolution. Nat Rev Microbiol 3:722–732. doi:10.1038/nrmicro1235 16138100

[11] Arora S, Li X, Hillhouse A, Konganti K, Little SV, Lawhon SD, Threadgill D, Shelburne S, Hook M. 2020. Staphylococcus epidermidis MSCRAMM SesJ is encoded in composite islands. mBio 11:e02911-19. doi:10.1128/mBio.02911-19 32071265 PMC7029136

[12] Manzer HS, Nobbs AH, Doran KS. 2020. The multifaceted nature of streptococcal antigen I/II proteins in colonization and disease pathogenesis. Front Microbiol 11:602305. doi:10.3389/fmicb.2020.602305 33329493 PMC7732690

[13] Pickering AC, Fitzgerald JR. 2020. The role of gram-positive surface proteins in bacterial Niche- and host-specialization. Front Microbiol 11:594737. doi:10.3389/fmicb.2020.594737 33193271 PMC7658395

[14] Arora S., Gordon J, Hook M. 2021. Collagen binding proteins of gram-positive pathogens. Front Microbiol 12:628798. doi:10.3389/fmicb.2021.628798 33613497 PMC7893114

[15] Arora S. 2017. S. epidermidis cell wall anchored proteins located in composite SCCmec islands. Texas A & M University.

[16] Antoniak S. 2018. The coagulation system in host defense. Res Pract Thromb Haemost 2:549–557. doi:10.1002/rth2.12109 30046760 PMC6046589

[17] Liesenborghs L, Verhamme P, Vanassche T. 2018. Staphylococcus aureus, master manipulator of the human hemostatic system. J Thromb Haemost 16:441–454. doi:10.1111/jth.13928 29251820

[18] Thomas S, Arora S, Liu W, Churion K, Wu Y, Höök M. 2021. Vhp is a fibrinogen-binding protein related to vWbp in Staphylococcus aureus. mBio 12:e0116721. doi:10.1128/mBio.01167-21 34340548 PMC8406236

[19] Olp MD, Kalous KS, Smith BC. 2020. ICEKAT: an interactive online tool for calculating initial rates from continuous enzyme kinetic traces. BMC Bioinformatics 21:186. doi:10.1186/s12859-020-3513-y 32410570 PMC7222511

[20] Almagro Armenteros JJ, Tsirigos KD, Sønderby CK, Petersen TN, Winther O, Brunak S, von Heijne G, Nielsen H. 2019. SignalP 5.0 improves signal peptide predictions using deep neural networks. Nat Biotechnol 37:420–423. doi:10.1038/s41587-019-0036-z 30778233

[21] Krogh A, Larsson B, von Heijne G, Sonnhammer EL. 2001. Predicting transmembrane protein topology with a hidden Markov model: application to complete genomes. J Mol Biol 305:567–580. doi:10.1006/jmbi.2000.4315 11152613

[22] Marchler-Bauer A, Bryant SH. 2004. CD-Search: protein domain annotations on the fly. Nucleic Acids Res 32:W327–31. doi:10.1093/nar/gkh454 15215404 PMC441592

[23] Kelley LA, Mezulis S, Yates CM, Wass MN, Sternberg MJE. 2015. The Phyre2 web portal for protein modeling, prediction and analysis. Nat Protoc 10:845–858. doi:10.1038/nprot.2015.053 25950237 PMC5298202

[24] Yu NY, Wagner JR, Laird MR, Melli G, Rey S, Lo R, Dao P, Sahinalp SC, Ester M, Foster LJ, Brinkman FSL. 2010. PSORTb 3.0: improved protein subcellular localization prediction with refined localization subcategories and predictive capabilities for all prokaryotes. Bioinformatics 26:1608–1615. doi:10.1093/bioinformatics/btq249 20472543 PMC2887053

[25] Jumper J, Evans R, Pritzel A, Green T, Figurnov M, Ronneberger O, Tunyasuvunakool K, Bates R, Žídek A, Potapenko A, et al.. 2021. Highly accurate protein structure prediction with AlphaFold. Nature 596:583–589. doi:10.1038/s41586-021-03819-2 34265844 PMC8371605

[26] Pettersen EF, Goddard TD, Huang CC, Meng EC, Couch GS, Croll TI, Morris JH, Ferrin TE. 2021. UCSF ChimeraX : structure visualization for researchers, educators, and developers . Protein Sci 30:70–82. doi:10.1002/pro.3943 32881101 PMC7737788

[27] Gillespie JJ, Wattam AR, Cammer SA, Gabbard JL, Shukla MP, Dalay O, Driscoll T, Hix D, Mane SP, Mao C, Nordberg EK, Scott M, Schulman JR, Snyder EE, Sullivan DE, Wang C, Warren A, Williams KP, Xue T, Yoo HS, Zhang C, Zhang Y, Will R, Kenyon RW, Sobral BW. 2011. PATRIC: the comprehensive bacterial bioinformatics resource with a focus on human pathogenic species. Infect Immun 79:4286–4298. doi:10.1128/IAI.00207-11 21896772 PMC3257917

[28] Davis JJ, Wattam AR, Aziz RK, Brettin T, Butler R, Butler RM, Chlenski P, Conrad N, Dickerman A, Dietrich EM, et al.. 2020. The PATRIC bioinformatics resource center: expanding data and analysis capabilities. Nucleic Acids Res 48:D606–D612. doi:10.1093/nar/gkz943 31667520 PMC7145515

[29] Olson RD, Assaf R, Brettin T, Conrad N, Cucinell C, Davis JJ, Dempsey DM, Dickerman A, Dietrich EM, Kenyon RW, et al.. 2023. Introducing the bacterial and viral bioinformatics resource center (BV-BRC): a resource combining PATRIC, IRD and ViPR. Nucleic Acids Res 51:D678–D689. doi:10.1093/nar/gkac1003 36350631 PMC9825582

[30] Alikhan N-F, Petty NK, Ben Zakour NL, Beatson SA. 2011. BLAST ring image generator (BRIG): simple prokaryote genome comparisons. BMC Genomics 12:402. doi:10.1186/1471-2164-12-402 21824423 PMC3163573

[31] Kohler V, Keller W, Grohmann E. 2019. Regulation of Gram-Positive conjugation. Front Microbiol 10:1134. doi:10.3389/fmicb.2019.01134 31191478 PMC6540685

[32] Li X, Arias CA, Aitken SL, Galloway Peña J, Panesso D, Chang M, Diaz L, Rios R, Numan Y, Ghaoui S, DebRoy S, Bhatti MM, Simmons DE, Raad I, Hachem R, Folan SA, Sahasarabhojane P, Kalia A, Shelburne SA. 2018. Clonal emergence of invasive multidrug-resistant Staphylococcus epidermidis deconvoluted via a combination of whole-genome sequencing and microbiome analyses. Clin Infect Dis 67:398–406. doi:10.1093/cid/ciy089 29546356 PMC6051468

[33] Patti JM, Höök M. 1994. Microbial adhesins recognizing extracellular matrix macromolecules. Curr Opin Cell Biol 6:752–758. doi:10.1016/0955-0674(94)90104-x 7833055

[34] Hook M, Foster TJ. 2021. Editorial: cell surface proteins of Gram-Positive pathogenic bacteria. Front Microbiol 12:681880. doi:10.3389/fmicb.2021.681880 34093507 PMC8175636

[35] Foster TJ, Geoghegan JA, Ganesh VK, Höök M. 2014. Adhesion, invasion and evasion: the many functions of the surface proteins of Staphylococcus aureus. Nat Rev Microbiol 12:49–62. doi:10.1038/nrmicro3161 24336184 PMC5708296

[36] Flick MJ, Du X, Prasad JM, Raghu H, Palumbo JS, Smeds E, Höök M, Degen JL. 2013. Genetic elimination of the binding motif on fibrinogen for the S. aureus virulence factor ClfA improves host survival in septicemia. Blood 121:1783–1794. doi:10.1182/blood-2012-09-453894 23299312 PMC3591798

[37] Pietrocola G, Nobile G, Gianotti V, Zapotoczna M, Foster TJ, Geoghegan JA, Speziale P. 2016. Molecular interactions of human plasminogen with fibronectin-binding protein B (FnBPB), a fibrinogen/fibronectin-binding protein from Staphylococcus aureus. J Biol Chem 291:18148–18162. doi:10.1074/jbc.M116.731125 27387503 PMC5000064

[38] Jeney A, Barrie SE, Taylor GA, Newell DR, Harrap KR, Szabolcs A, Lapis K, Otvös L. 1986. 5-Ethyl-2’-deoxyuridine: an explanation for its lack of cytotoxic action in vivo. Eur J Cancer Clin Oncol 22:557–562. doi:10.1016/0277-5379(86)90043-x 3770027

[39] Friberger P, Knös M, Gustavsson S, Aurell L, Claeson G. 1978. Methods for determination of plasmin, antiplasmin and plasminogen by means of substrate S-2251. Haemostasis 7:138–145. doi:10.1159/000214252 149046

[40] Chapin JC, Hajjar KA. 2015. Fibrinolysis and the control of blood coagulation. Blood Rev 29:17–24. doi:10.1016/j.blre.2014.09.003 25294122 PMC4314363

[41] Davis SL, Gurusiddappa S, McCrea KW, Perkins S, Höök M. 2001. SdrG, a fibrinogen-binding bacterial adhesin of the microbial surface components recognizing adhesive matrix molecules subfamily from Staphylococcus epidermidis, targets the thrombin cleavage site in the Bbeta chain. J Biol Chem 276:27799–27805. doi:10.1074/jbc.M103873200 11371571

[42] Bhattacharya S, Ploplis VA, Castellino FJ. 2012. Bacterial plasminogen receptors utilize host plasminogen system for effective invasion and dissemination. J Biomed Biotechnol 2012:482096. doi:10.1155/2012/482096 23118509 PMC3477821

[43] Guo Y, Li J, Hagström E, Ny T. 2011. Beneficial and detrimental effects of plasmin(ogen) during infection and sepsis in mice. PLoS ONE 6:e24774. doi:10.1371/journal.pone.0024774 21931850 PMC3171470

[44] Keragala CB, Medcalf RL. 2021. Plasminogen: an enigmatic zymogen. Blood 137:2881–2889. doi:10.1182/blood.2020008951 33735914

[45] Grella DK, Castellino FJ. 1997. Activation of human plasminogen by staphylokinase. direct evidence that preformed plasmin is necessary for activation to occur. Blood 89:1585–1589. doi:10.1182/blood.V89.5.1585 9057640

[46] Loof TG, Deicke C, Medina E. 2014. The role of coagulation/fibrinolysis during Streptococcus pyogenes infection. Front Cell Infect Microbiol 4:128. doi:10.3389/fcimb.2014.00128 25309880 PMC4161043

[47] Draxler DF, Sashindranath M, Medcalf RL. 2017. Plasmin: a modulator of immune function. Semin Thromb Hemost 43:143–153. doi:10.1055/s-0036-1586227 27677178

[48] Pryzdial ELG, Leatherdale A, Conway EM. 2022. Coagulation and complement: key innate defense participants in a seamless web. Front Immunol 13:918775. doi:10.3389/fimmu.2022.918775 36016942 PMC9398469

[49] Vago JP, Zaidan I, Perucci LO, Brito LF, Teixeira LC, Silva CMS, Miranda TC, Melo EM, Bruno AS, Queiroz-Junior CM, Sugimoto MA, Tavares LP, Grossi LC, Borges IN, Schneider AH, Baik N, Schneider AH, Talvani A, Ferreira RG, Alves-Filho JC, Nobre V, Teixeira MM, Parmer RJ, Miles LA, Sousa LP. 2023. Plasmin and plasminogen prevent sepsis severity by reducing neutrophil extracellular traps and systemic inflammation. JCI Insight 8:e166044. doi:10.1172/jci.insight.166044 36917195 PMC10243804

