# Supplementary material for: Staphylococcus epidermidis ST2 strains associated with bloodstream infections contain a unique mobile genetic element encoding a plasmin inhibitor
Source: mBio. 2024 Nov 19;15(12):e01907-24. doi: 10.1128/mbio.01907-24 (PMC11633098; doi:10.1128/mbio.01907-24)
Supplement: Supplemental material — Supplemental text and figures. [file mbio.01907-24-s0001.docx]

***Staphylococcus epidermidis* ST2 strains associated with bloodstream infections contain a unique mobile genetic element encoding a plasmin inhibitor.**

Amy A. Gomez^1Δ^, Clara Kjerfve^1ɸΔ^, Minseo Choi^1^, Wen Liu^1^, Kelly Churion^1^, Sheila Thomas^1#^, Holger Rohde^2^, Sam Shelburne^3^, Jon T. Skare^4^, Magnus Hook^1^_,_ Srishtee Arora^1*^

^1^ Center for Infectious and Inflammatory Diseases, Institute of Biosciences and Technology, Texas A&M Health Science Center, Houston, Texas, USA

^2^ Institute for Medical Microbiology, Virology and Hygiene, University Medical Center Hamburg-Eppendorf, Hamburg, Germany

^3^Department of Infectious Diseases, Infection Control, and Employee Health, Division of Internal Medicine, MD Anderson Cancer Center, Houston, Texas, USA

^4^Department of Microbial Pathogenesis and Immunology, School of Medicine, Texas A&M Health Science Center, Bryan/College Station, Texas, USA

^Δ^ Amy A. Gomez and Clara Kjerfve are equal shared authors. Author order was determined both alphabetically and in order of increased seniority.

^ɸ^ Current address: Department of Chemistry and Biochemistry, University of South Carolina, Columbia, South Carolina

^#^  Current address: Department of Microbiology and Immunology, East Carolina University, Greenville, North Carolina, USA.

* Corresponding authors: Magnus Hook mhook@tamu.edu

**SUPPLEMENTAL RESULTS**

**Characterization of putative Integrated Conjugative Element Sepi-ST2**

The encoded ORFs in pICE-Sepi-ST2 can be divided into families like conjugation machinery, DNA binding proteins, and surface adhesins. Conjugation machinery and DNA binding are covered here. For additional details on surface adhesions, see the main text.

*Conjugation machinery.*

pICE-Sepi-ST2 produces two ATPases encoded by ORF 1490 and 1495. ORF 1490 produces ATPase like VirB4 (COG3451, e-value 1.89e-07) at positions 207-620. 1490 ATPase is a 649 amino acid (aa) long intracellular protein with walker A (GKMGMGKS) and B (MFFIDE) motifs (**Figure 2B, Figure S1**). ORF 1495 encodes for 859 aa long protein-like to VirD4-like ATPase (cl29730, e value – 1.14e-20) at positions 137-695. ORF 1495 ATPase has two N-terminal transmembrane helices and a 742 aa long extracellular domain that contains the predicted ATPase activity along with walker A (GTSRSGKG) and B (YPIYNE) motifs (**Figure 2B, Figure S1**).

pICE-Sepi-ST2 also encodes for VirB3-like, VirB6-like, and a VirB8-like proteins that form the transmembrane channel in Gram-positive bacteria. ORF1497 encodes for class gamma VirB8-like protein [1, 2], which contains a 575 aa long N-terminal domain, a single transmembrane helix, and a C-terminal cytoplasmic domain (**Figure 2B, Figure S1**). ORF 1502 protein is a 292 amino acid long VirB6-like protein [1, 2] containing 6 transmembrane helices spanned throughout the protein (**Figure 2B, Figure S1**). 1492 is a VirB3-like protein [1, 2] with a cytoplasmic N-terminus, two transmembrane helices, and a 33-long C-terminus cytoplasmic tail (**Figure 2B, Figure S1**).

In addition to VirB3-like, VirB6-like, and VirB8-like proteins, pICE-Sepi-ST2 also encodes for three more transmembrane proteins with no significant similarity to known proteins of other Gram-positive type IV secretion systems. These include ORF 1488, ORF 1494, and ORF 1498. ORF 1488 and OFR 1498 are small proteins with 200 and 100 aa each, respectively. 1488 has a cytoplasmic N-terminus, a single transmembrane helix, and a 173-a-long extracellular region (**Figure 2B**). On the other hand, 1498 has an extracellular 42-aa long N-terminal tail and two transmembrane helices (**Figure 2B, Figure S1**). 1494 is relatively large, with 765 residues. It has a small N-terminal extracellular region, four transmembrane helices, and a 392 aa extracellular C-terminal domain. Transmembrane helix 3 and 4 of 1494 are connected by 156 aa regions (**Figure 2B, Figure S1**). The functions of the extracellular domains present in these three proteins have yet to be discovered.

Conjugation systems in Gram-positive bacteria contain a peptidoglycan hydrolase that breaks down the cell wall [1, 2]. In pICE-Sepi-ST2, peptidoglycan hydrolase is encoded by ORF 1489. It has two N-terminal transmembrane helices and two peptidoglycan degrading domains: cysteine, histidine-dependent amidohydrolases/peptidases (CHAP) domain, and a soluble lytic transglucosylase (SLT) domain.

*DNA binding proteins*

pICE-Sepi-ST2 encodes two putative DNA binding proteins: relaxase and integrase. Relaxase is encoded by ORF 1483 and belongs to the MOB_P_ family as determined by the online MOBscan program [3]. Profile HMM for pICE relaxase is T4SS_MOBP2, including MOB_L_ relaxase prototype Rel_LS20_ of plasmid pLS20 [4, 5]. Integrase is a 359aa protein encoded by ORF1509 and shows similarity to integrase from *Bacillus subtilis* conjugative transposon ICEBs1 at residues 180 to 336 (cd01189, e value – 2.34e-23).

**REFERENCES**

1. Goessweiner-Mohr, N., et al., *Conjugative type IV secretion systems in Gram-positive bacteria.* Plasmid, 2013. **70**(3): p. 289-302.

2. Goessweiner-Mohr, N., et al., *Conjugation in Gram-Positive Bacteria.* Microbiol Spectr, 2014. **2**(4): p. PLAS-0004-2013.

3. Garcillán-Barcia, M.P., et al., *MOBscan: Automated Annotation of MOB Relaxases*, in *Horizontal Gene Transfer: Methods and Protocols*, F. de la Cruz, Editor. 2020, Springer US: New York, NY. p. 295-308.

4. Ramachandran, G., et al., *Discovery of a new family of relaxases in Firmicutes bacteria.* PLOS Genetics, 2017. **13**(2): p. e1006586.

5. Garcillán-Barcia, M.P., M.V. Francia, and F. de la Cruz, *The diversity of conjugative relaxases and its application in plasmid classification.* FEMS Microbiol Rev, 2009. **33**(3): p. 657-87.

6. Olson, R.D., et al., *Introducing the Bacterial and Viral Bioinformatics Resource Center (BV-BRC): a resource combining PATRIC, IRD and ViPR.* Nucleic Acids Res, 2023. **51**(D1): p. D678-D689.


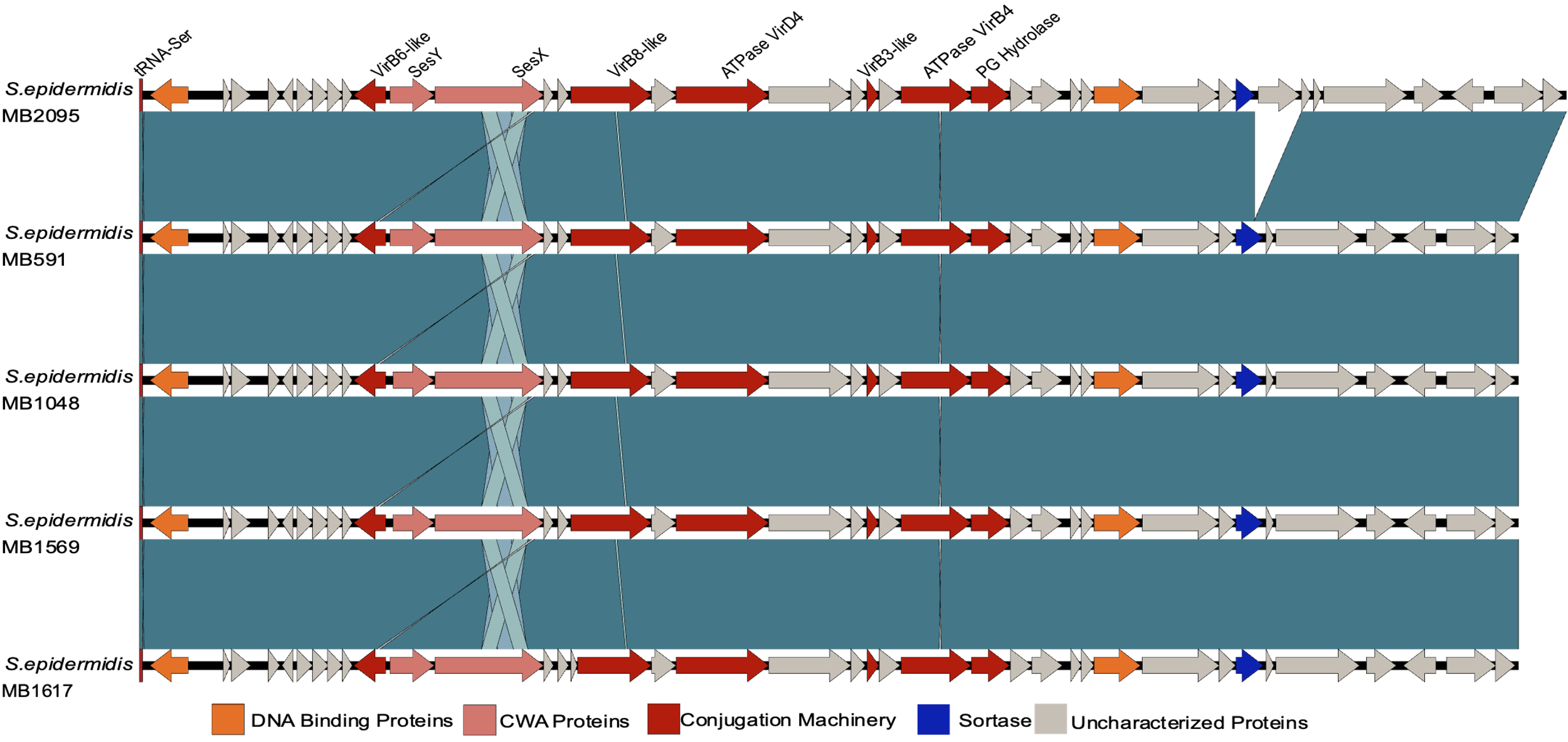


**Figure S1.** **Comparison of pICE in *S. epidermidis* ST2 blood isolates.** Schematic representation of pICE containing the sesX and sesY genes in S. epidermidis MB2095, MB591, MB1048, MB1569, and MB1617. The alignment was created by Easy Fig which uses BLAST results to show aligned regions between the region in light blue with a black border representing a region of at least 99% identity at the nucleic acid level [6]. Each ORF is represented by an arrow, with the direction of the arrow indicating the direction of transcription.


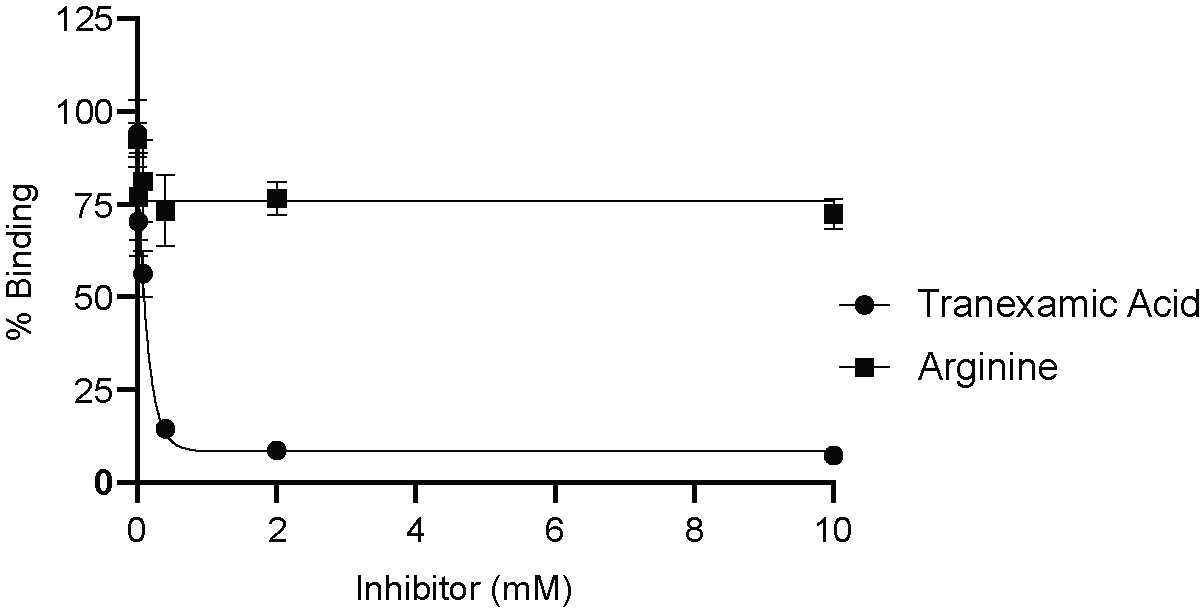


**Figure S2.** **Inhibition of SesY binding to plasminogen by tranexamic acid.** Inhibition of rSesY binding to immobilized Plg was assessed in varying concentrations of a lysine derivative, tranexamic acid (circle), and arginine (square). rSesY bound to Plg was detected with α -His Tag HRP conjugated antibody at a 1:3000 dilution. The presence of tranexamic acid at 2 µM decreased rSesY binds to Pln by 85%. Results from three biological replicates are presented. Error bars indicate the standard deviation (mean ± SD).


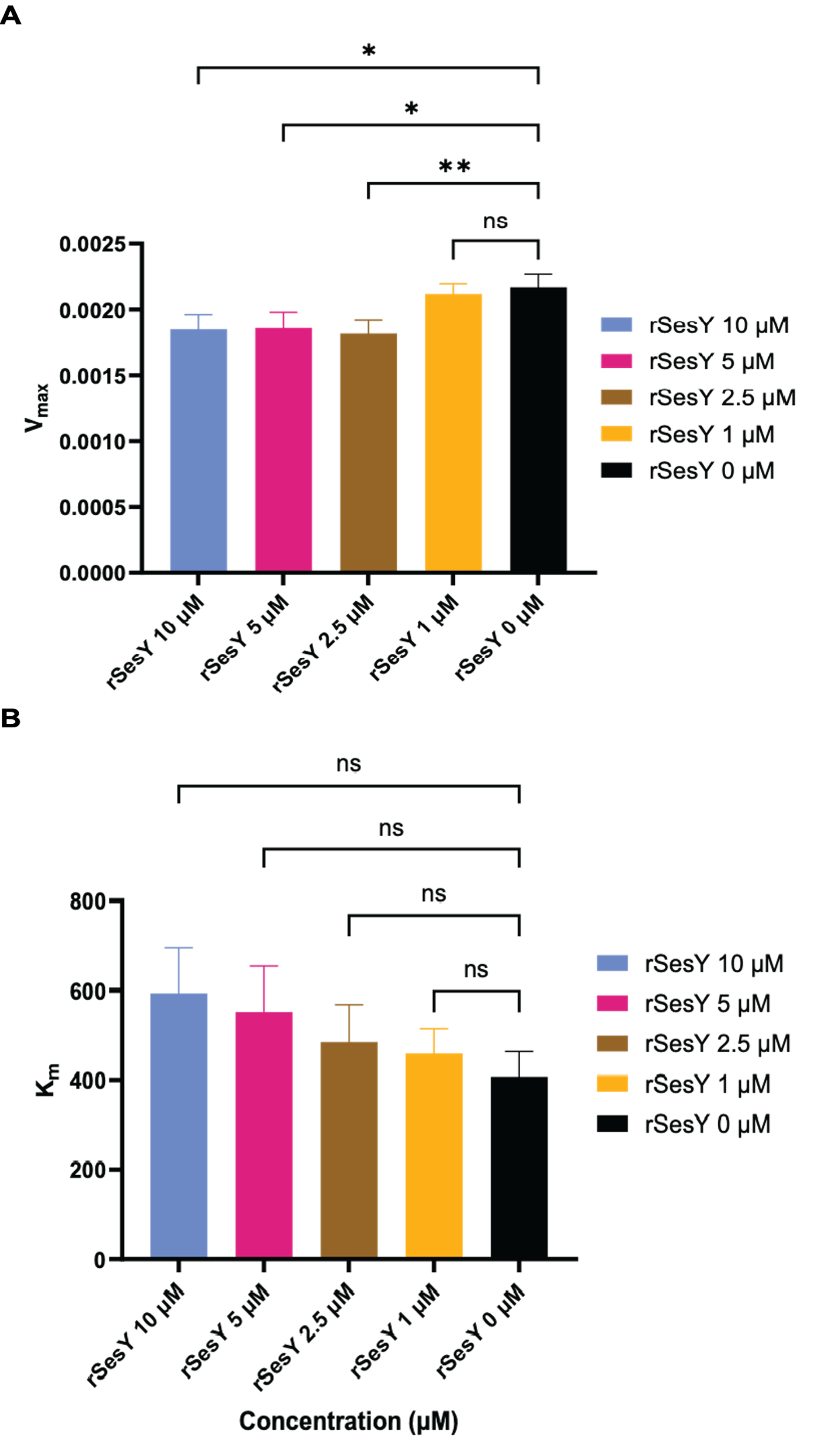


**Figure S3.** **V_max_ decreased, and K_max_ was unaffected in the presence of SesY.** V_max_ generated from Michaelis-Menten curves. Results from three biological replicates are represented. Error bars indicate the standard deviation (mean ± SD, *P < 0.01118), *P < 0.0142), **P < 0.0067).

**
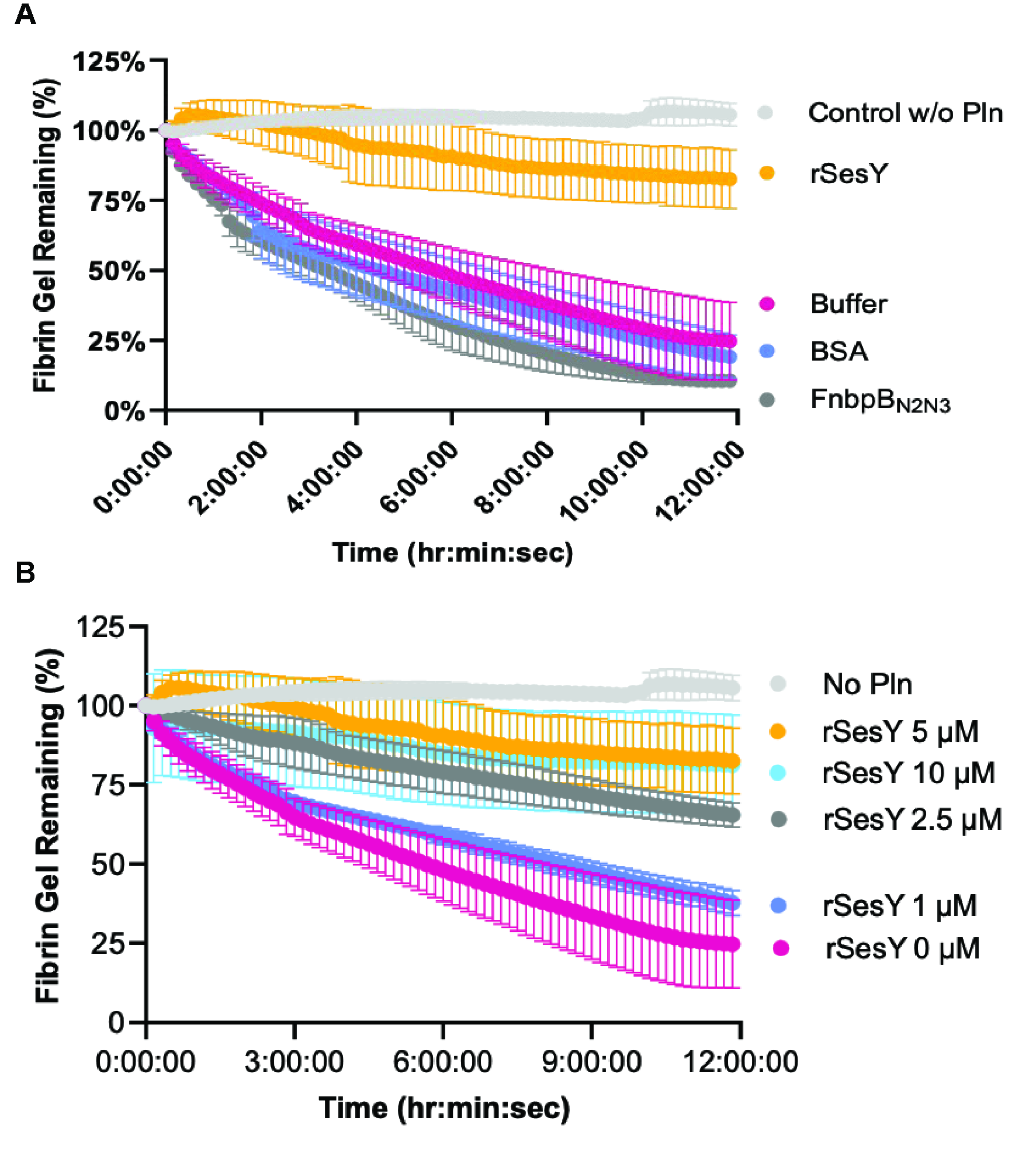
**

**Figure S4**. **rSesY_32-341_ inhibits the Pln-mediated degradation of fibrin.** (A) Degradation of fibrin was observed at 405 nm to measure turbidity for 12hrs using a kinetic cycle with readings every 5 mins in the presence of Pln and 5 µM of different proteins: FnbpB_N2N3_, rSesY_32-342,_ and BSA. Buffer control represents wells with 0 µM of additional protein. No Pln represents wells with pre-formed fibrin gel without the addition of Pln. (B) Degradation of fibrin was observed at 405 nm to measure turbidity for 12hrs using a kinetic cycle with readings every 5 mins in the presence of Pln and different concentrations of rSesY_32-341_ (0 µM, 1 µM, 2.5 µM, 5 µM, 10 µM). Buffer control represents wells with 0 µM of additional protein. No Pln represents wells with pre-formed fibrin gel without the addition of Pln. Results from three biological replicates are represented. Error bars indicate the standard deviation (mean ± SD).
